# Supplementary material for: TDCS over PPC or DLPFC does not improve visual working memory capacity
Source: Commun Psychol. 2024 Mar 11;2:20. doi: 10.1038/s44271-024-00067-8 (PMC11332112; doi:10.1038/s44271-024-00067-8)
Supplement: Supplementary file 1 — Supplementary Information [file 44271_2024_67_MOESM1_ESM.pdf]

Supplementary Information

TDCS over PPC or DLPFC does not improve visual working memory capacity

Shuangke Jiang, Myles Jones, and Claudia C. von Bastian

Department of Psychology and Neuroscience Institute, University of Sheffield, Sheffield, UK

Supplementary Notes 1: Blindness and Post-Stimulation Rating

To ensure participants were blind to the condition, we examined their self-reported guesses about which stimulation condition they thought they had received by running one-sample Wilcoxon tests, as the data violated the assumption of normality. For the sham condition, guessing accuracy was 52.08%, which was not significantly different from chance guessing of 50%, $V = 612.50$, $p = 0.777$, $r = 0.04$, $BF_{10} = 1/6.14 \pm 0.06\%$. This result confirms that overall participants were not able to differentiate the sham stimulation from active stimulations, indicating our blinding was effective in preventing placebo effects.

For each of the sessions you have completed you received either real or sham* tDCS.

Please circle the appropriate answer.

Which tDCS do you think you received during session 1?	Real / Sham / I am not sure
Which tDCS do you think you received during session 2?	Real / Sham / I am not sure
Which tDCS do you think you received during session 3?	Real / Sham / I am not sure

* The purpose of sham stimulation was to act as a control (placebo), ensuring participants experienced a similar itching feeling that receded over the first few seconds of active stimulation. Sham stimulation lasted for a few seconds from the initial time of the stimulation.

We also investigated the level of pain, attention, and fatigue after each stimulation. We found that only pain level after DLPFC stimulation (*M* = 1.83, *SD* = 1.10) was significantly higher than that after sham (*M* = 1.33, *SD* = 0.72), $t(47) = 3.07$, Bonferroni-adjusted $p = 0.011$, Cohen's $d = 0.44$, $BF_{10} = 9.29 \pm 0.00\%$. However, this did not affect their overall cognitive performance. Note that there was no significant difference between stimulation conditions regarding the levels of attention (PPC vs sham: Bonferroni-adjusted $p = 0.067$; DLPFC vs sham: Bonferroni-adjusted $p = 0.339$; PPC vs DLPFC: Bonferroni-adjusted $p = 1.000$) and fatigue (Bonferroni-adjusted *p* values were all 1.000).

Post Stimulation Ratings – Session 1 / session 2

Participant ID:_________

Please rate your "current" levels of pain, attention and fatigue.

Minimal Pain Maximal Pain

I------------------I------------------I------------------I------------------I------------------I------------------I

1 2 3 4 5 6 7

Poorest Attention Maximal Attention

I------------------I------------------I------------------I------------------I------------------I------------------I

1 2 3 4 5 6 7

Minimal Fatigue						Maximal Fatigue
I-------------------I	-------------------I	-------------------I	-------------------I	-------------------I	-------------------I	
1	2	3	4	5	6	7

Supplementary Notes 2: Adverse tDCS Effects

Overall, the current tDCS setup did not largely lead to any severe adverse effect, which indicates the safety of the montage and paradigms (Supplementary Table 1). Of all 144 experimental sessions, the most common self-reported symptoms, that were at least possibly related to tDCS, were tingling (58.33%) and itching (45.83%) sensations, followed by skin redness (30.56%) and burning sensations (27.78%).

tDCS Adverse Effects Questionnaire:

Session:

ID:

Do you experience any of the following symptoms or side-effects?	Enter a value (1–4) in the space below (1, absent; 2, mild; 3, moderate; 4, severe)	If present: Is this related to tDCS? (1, none; 2, remote; 3, possible; 4, probable; 5, definite)
Headache		
Neck pain		
Scalp pain		
Tingling		
Itching		

Burning sensation		
Skin redness		
Sleepiness		
Trouble concentrating		
Acute mood change		
Others (specify)		

Supplementary Table 1

Self-Reported Adverse Effects After Stimulation

Symptom	General (%)	TDCS-Related (%)
Headache	15.28	10.42
Neck pain	5.56	2.08
Scalp pain	13.19	12.50
Tingling	61.81	58.33
Itching	52.08	45.83
Burning sensation	29.17	27.78
Skin redness	34.72	30.56
Sleepiness	36.11	13.19
Trouble concentrating	14.58	7.64
Acute mood change	4.86	3.47

Note. A total of 144 sessions (three sessions for each participant). General: percentage of reported mild to severe symptom (2-mild, 3-moderate, 4-severe); TDCS-Related: percentage of reported symptom that is at least possibly related to stimulation (3-possible, 4-probable, 5-definite).

Supplementary Notes 3: Head Size

Supplementary Table 2

Individual Head Model

Participant	NI	TT	C
1	38.00	35.00	61.50
2	34.00	37.00	55.00
3	34.00	37.00	56.50
4	32.00	32.50	54.00
5	36.00	35.00	56.00
6	37.00	37.00	59.00
7	35.00	33.50	55.00
8	36.00	34.00	58.00
9	35.00	34.00	55.00
10	36.00	36.50	56.00
11	33.00	36.00	55.50
12	35.00	34.50	57.80
13	34.00	36.50	56.50
14	36.50	37.50	58.00
15	37.00	36.50	60.50
16	32.50	32.50	54.00
17	33.00	36.00	57.00
18	32.00	33.00	54.00
19	36.00	37.50	58.00
20	38.00	37.00	60.00
21	35.50	35.50	57.00
22	33.00	33.00	54.00
23	35.50	35.00	58.00
24	33.50	35.00	57.50
25	32.00	35.00	53.00
26	34.00	37.00	55.00
27	37.00	36.00	56.50
28	35.00	35.00	55.00
29	34.00	35.00	56.00
30	32.00	34.00	51.50
31	35.00	34.00	57.00
32	32.00	34.00	54.50
33	36.00	37.00	56.00
34	32.00	34.00	53.00
35	34.00	35.00	52.00
36	32.00	35.00	53.50
37	35.00	38.00	56.50
38	34.00	35.00	56.00
39	36.00	37.00	58.00
40	35.00	37.00	55.50
41	32.00	34.00	53.50
42	37.00	35.00	59.00

Supplementary Table 2

Individual Head Model

Participant	NI	TT	C
43	36.00	35.00	56.50
44	33.00	35.00	54.00
45	36.00	36.00	56.50
46	34.00	35.00	53.50
47	35.00	36.00	58.00
48	34.00	33.00	55.00

Note. NI = length between nasion and inion (cm): TT = length from left tragus to right tragus (cm); C = circumference length (cm).

Supplementary Notes 4: The AIC and BIC Values of Each Participant in All Conditions

Supplementary Table 3

Differences in Relative Akaike Information Criterion Values (ΔAIC)

Participant	Sham			PPC			DLPFC		
	Set size								
	2	4	6	2	4	6	2	4	6
1	-2.00	2.72	-2.00	-2.00	10.61	1.10	-2.01	7.92	5.65
2	-2.00	12.18	11.40	-2.00	4.04	3.65	-2.00	6.36	5.64
3	-2.06	-2.00	0.11	-2.25	-1.34	3.03	-2.00	-2.00	0.48
4	2.17	3.14	-0.27	-0.52	-2.00	14.15	-2.00	0.08	-0.35
5	-2.00	-1.74	-1.20	-2.00	-1.96	1.78	-2.00	-0.88	4.65
6	-2.00	-1.69	2.61	-2.00	2.10	-0.53	-2.00	2.07	-0.69
7	-2.00	1.13	-2.00	2.93	-1.84	-1.14	2.64	1.67	-1.80
8	-1.55	0.94	-0.99	-1.14	1.00	5.69	-2.00	0.02	-2.00
9	2.21	2.19	-1.14	-1.45	2.37	-2.00	-2.00	2.76	-0.72
10	-2.00	-1.81	-0.93	-2.00	-0.69	8.61	5.36	0.97	-1.04
11	-1.87	3.82	-1.70	-2.00	-0.83	-1.65	-2.00	3.94	-1.75
12	-2.00	-1.79	-1.99	-2.00	-0.71	18.69	-2.07	-1.33	-1.68
13	1.96	0.53	5.94	-1.66	6.71	0.11	1.20	4.83	1.38
14	-2.00	-1.96	-1.08	-1.16	-0.91	-1.46	-2.00	-0.32	-0.34
15	-2.00	10.32	10.30	-2.00	0.94	16.73	0.77	7.78	28.49
16	4.75	-2.00	-1.91	2.24	-2.00	0.40	-0.29	-2.00	6.84
17	-1.71	-2.00	-2.00	-2.00	7.14	1.83	-1.73	-1.89	-1.94
18	0.91	1.93	1.06	-2.00	0.89	1.25	0.22	5.11	7.25
19	-2.00	-2.00	-1.32	-2.00	1.43	-1.84	-2.00	0.19	-1.49
20	-2.00	-2.00	-2.00	-2.00	2.02	0.78	-2.00	1.52	-0.87
21	-2.00	-0.26	-0.17	-2.00	-1.22	5.03	-2.00	2.58	-2.00
22	-1.81	-1.83	-2.00	3.18	-2.00	-2.00	-2.00	6.07	1.65
23	-2.00	0.18	-0.10	2.60	1.10	1.61	-2.00	6.06	3.12
24	-2.00	3.73	-0.10	-2.00	-2.00	-2.00	-2.01	-1.37	12.07
25	-2.00	0.47	1.92	-2.00	7.94	4.18	-2.00	1.71	3.35
26	-2.01	7.04	5.67	-2.00	-2.00	7.59	-2.00	-0.96	1.29
27	-2.00	8.34	12.27	-2.00	-0.92	9.94	-2.00	0.22	-2.00
28	7.34	6.74	2.95	-2.00	-1.02	4.87	-2.00	-2.00	0.27
29	-2.00	-2.00	-2.00	-2.00	-2.00	1.03	-2.00	-0.05	-2.00
30	-1.23	-0.39	7.61	-1.96	10.18	5.79	-2.00	2.80	7.68
31	-1.12	-1.44	0.54	-1.47	-1.73	5.46	-2.00	-0.02	-0.41
32	-2.00	0.17	-1.98	-2.00	-2.00	-1.94	-2.00	-0.71	1.12
33	-1.35	-2.00	-2.00	-2.00	7.70	-1.82	-2.00	1.19	-1.07
34	-2.00	-1.44	-2.00	-2.00	-2.00	7.48	-1.99	-2.00	1.42
35	2.55	-1.97	-1.57	2.31	-0.89	2.84	-2.00	-1.77	-2.00
36	3.97	-1.34	-1.88	-2.00	9.42	2.22	1.35	1.32	-0.96
37	-0.39	7.62	-1.14	-2.06	0.76	2.46	-0.75	2.48	0.92
38	-0.38	3.43	1.23	-2.00	-1.83	-2.00	9.43	-0.48	1.72
39	-2.01	0.62	0.10	-2.11	-2.00	-1.08	-2.00	-2.00	-0.77
40	-2.00	-2.00	-2.00	-2.01	-0.44	-2.00	-2.00	4.28	3.66

Supplementary Table 3

Differences in Relative Akaike Information Criterion Values (ΔAIC)

Participant	Sham			PPC			DLPFC		
	Set size								
	2	4	6	2	4	6	2	4	6
41	-1.86	-1.35	-1.78	-2.00	-0.34	0.73	3.27	-2.00	1.97
42	-2.00	-0.75	-1.86	-2.01	9.60	3.78	-2.00	4.18	5.34
43	-2.00	1.03	2.22	3.27	-1.69	0.85	-1.93	-1.88	2.38
44	-2.00	-2.00	-1.33	-2.02	-1.77	-0.78	-1.14	-1.74	-2.00
45	-1.98	11.99	2.68	-2.12	0.41	9.60	-2.00	-0.79	4.17
46	-2.00	3.93	-0.46	4.81	8.39	2.08	3.88	1.25	-1.90
47	-2.00	3.27	-2.00	-2.01	-1.66	-1.96	-2.00	-2.00	2.28
48	-1.24	-2.00	1.03	-2.00	0.91	-2.00	1.25	-1.38	-1.92

Note. Negative values favour SMM while positive values favour SM. The lower value represents a better model fit. SMM = standard mixture model; SM = swap model.

Supplementary Table 4

Differences in Relative Bayesian Information Criterion Values (ΔBIC)

Participant	Sham			PPC			DLPFC		
	Set size								
	2	4	6	2	4	6	2	4	6
1	-6.63	-1.91	-6.63	-6.63	5.98	-3.53	-6.63	3.30	1.02
2	-6.63	7.55	6.78	-6.63	-0.59	-0.98	-6.63	1.73	1.01
3	-6.69	-6.63	-4.52	-6.88	-5.96	-1.60	-6.63	-6.63	-4.15
4	-2.46	-1.48	-4.90	-5.15	-6.63	9.53	-6.63	-4.54	-4.97
5	-6.63	-6.36	-5.83	-6.63	-6.58	-2.84	-6.63	-5.50	0.02
6	-6.63	-6.31	-2.02	-6.63	-2.52	-5.15	-6.63	-2.56	-5.32
7	-6.63	-3.50	-6.63	-1.69	-6.47	-5.77	-1.98	-2.96	-6.43
8	-6.17	-3.68	-5.62	-5.76	-3.63	1.06	-6.63	-4.61	-6.63
9	-2.41	-2.43	-5.77	-6.08	-2.25	-6.63	-6.63	-1.87	-5.34
10	-6.63	-6.44	-5.56	-6.63	-5.32	3.99	0.74	-3.66	-5.66
11	-6.49	-0.80	-6.32	-6.63	-5.46	-6.28	-6.63	-0.68	-6.37
12	-6.63	-6.41	-6.62	-6.63	-5.33	14.07	-6.70	-5.95	-6.31
13	-2.67	-4.10	1.32	-6.28	2.09	-4.52	-3.43	0.21	-3.25
14	-6.63	-6.59	-5.70	-5.79	-5.54	-6.09	-6.63	-4.94	-4.96
15	-6.63	5.69	5.67	-6.63	-3.69	12.10	-3.86	3.15	23.87
16	0.13	-6.63	-6.53	-2.38	-6.63	-4.23	-4.92	-6.63	2.22
17	-6.33	-6.62	-6.63	-6.63	2.51	-2.80	-6.36	-6.51	-6.56
18	-3.72	-2.70	-3.57	-6.63	-3.74	-3.37	-4.40	0.48	2.62
19	-6.63	-6.63	-5.94	-6.63	-3.19	-6.47	-6.63	-4.43	-6.11
20	-6.63	-6.62	-6.62	-6.63	-2.61	-3.84	-6.63	-3.10	-5.50

Supplementary Table 4

Differences in Relative Bayesian Information Criterion Values (ΔBIC)

Participant	Sham			PPC			DLPFC		
	Set size								
	2	4	6	2	4	6	2	4	6
21	-6.63	-4.88	-4.80	-6.63	-5.84	0.41	-6.63	-2.04	-6.63
22	-6.43	-6.46	-6.63	-1.44	-6.63	-6.63	-6.63	1.44	-2.97
23	-6.63	-4.44	-4.72	-2.03	-3.52	-3.01	-6.63	1.44	-1.50
24	-6.63	-0.89	-4.72	-6.63	-6.63	-6.63	-6.63	-5.99	7.45
25	-6.63	-4.15	-2.70	-6.63	3.31	-0.44	-6.63	-2.92	-1.28
26	-6.63	2.41	1.04	-6.63	-6.63	2.97	-6.63	-5.58	-3.34
27	-6.63	3.72	7.65	-6.63	-5.54	5.31	-6.63	-4.41	-6.63
28	2.71	2.11	-1.67	-6.63	-5.65	0.25	-6.63	-6.63	-4.36
29	-6.63	-6.63	-6.63	-6.63	-6.62	-3.60	-6.63	-4.68	-6.62
30	-5.85	-5.02	2.98	-6.59	5.56	1.17	-6.63	-1.83	3.06
31	-5.75	-6.06	-4.09	-6.10	-6.36	0.84	-6.63	-4.64	-5.03
32	-6.63	-4.46	-6.60	-6.63	-6.63	-6.57	-6.63	-5.33	-3.51
33	-5.98	-6.63	-6.63	-6.63	3.07	-6.44	-6.63	-3.44	-5.69
34	-6.63	-6.07	-6.63	-6.63	-6.63	2.86	-6.62	-6.62	-3.21
35	-2.07	-6.60	-6.20	-2.32	-5.52	-1.79	-6.62	-6.39	-6.63
36	-0.66	-5.97	-6.50	-6.63	4.80	-2.41	-3.27	-3.30	-5.58
37	-5.01	3.00	-5.76	-6.68	-3.86	-2.17	-5.37	-2.14	-3.70
38	-5.01	-1.19	-3.40	-6.63	-6.46	-6.63	4.81	-5.11	-2.91
39	-6.63	-4.00	-4.53	-6.74	-6.63	-5.70	-6.63	-6.63	-5.40
40	-6.63	-6.63	-6.63	-6.63	-5.06	-6.63	-6.63	-0.35	-0.96
41	-6.49	-5.97	-6.40	-6.63	-4.97	-3.90	-1.36	-6.63	-2.66
42	-6.63	-5.37	-6.48	-6.64	4.97	-0.84	-6.63	-0.44	0.71
43	-6.63	-3.60	-2.41	-1.35	-6.32	-3.78	-6.56	-6.51	-2.24
44	-6.63	-6.63	-5.95	-6.65	-6.40	-5.40	-5.76	-6.36	-6.63
45	-6.60	7.36	-1.95	-6.75	-4.21	4.97	-6.63	-5.41	-0.45
46	-6.63	-0.69	-5.08	0.19	3.77	-2.54	-0.75	-3.37	-6.53
47	-6.63	-1.36	-6.63	-6.63	-6.29	-6.58	-6.63	-6.63	-2.34
48	-5.87	-6.63	-3.60	-6.63	-3.72	-6.63	-3.38	-6.01	-6.54

Note. Negative values favour SMM while positive values favour SM. The lower value represents a better model fit. SMM = standard mixture model; SM = swap model.

Supplementary Notes 5: Descriptive Statistics of Performance After Each Stimulation

Supplementary Table 5

Descriptive Statistics of Performance After Each Stimulation

Variable	Capacity (*K*)			Precision (SD^{-1})		
	M	*SD*	CI	*M*	*SD*	CI
Sham						
Set size 2	1.93	0.07	[1.91, 1.95]	0.08	0.02	[0.08, 0.09]
Set size 4	2.84	0.63	[2.66, 3.03]	0.06	0.01	[0.06, 0.07]
Set size 6	3.10	0.98	[2.81, 3.38]	0.06	0.01	[0.06, 0.06]
PPC						
Set size 2	1.92	0.09	[1.89, 1.94]	0.08	0.02	[0.08, 0.09]
Set size 4	2.84	0.74	[2.62, 3.05]	0.06	0.01	[0.06, 0.07]
Set size 6	2.96	1.14	[2.63, 3.29]	0.06	0.01	[0.06, 0.06]
DLPFC						
Set size 2	1.91	0.11	[1.88, 1.94]	0.08	0.02	[0.08, 0.09]
Set size 4	2.79	0.70	[2.58, 2.99]	0.06	0.01	[0.06, 0.06]
Set size 6	3.10	1.07	[2.79, 3.41]	0.06	0.02	[0.05, 0.06]

Note. Capacity ranges from 0 to the set size; precision ranges from 0 to ∞. *M* = mean; *SD* = standard deviation; CI = 95% confidence interval on the mean value.

Supplementary Notes 6: Posterior Distribution and Sensitivity Analyses for ANOVAs

Supplementary Table 6

Summary of Posterior Distribution for ANOVAs Interactions

Set size	PPC				DLPFC			
	M	*SD*	*LB*	*UB*	*M*	*SD*	*LB*	*UB*
2	0.01	0.04	-0.06	0.09	-0.01	0.04	-0.09	0.06
4	0.03	0.04	-0.04	0.11	-0.03	0.04	-0.11	0.04
6	-0.05	0.04	-0.13	0.03	0.05	0.04	-0.03	0.13
2	-0.00	0.00	-0.00	0.00	0.00	0.00	-0.00	0.00
4	0.00	0.00	-0.00	0.00	-0.00	0.00	-0.00	0.00
6	0.00	0.00	-0.00	0.00	-0.00	0.00	-0.00	0.00

Note. M = mean; *SD* = standard deviation; *LB* = lower bound of 95% credibility interval; *UB* = upper bound of 95% credibility interval.

Supplementary Table 7

Sensitivity of ANOVAs Statistics

Effect	Prior			
	Smaller		Larger	
	BF_{10}	Error (%)	BF_{10}	Error (%)
ST	1/6.74	2.10	1/6.90	2.79
SS	1/21.37	0.79	1/21.35	0.78
ST x SS	1/6.24	2.66	1/8.36	23.29
ST	1/7.35	1.18	1/7.34	1.26
SS	1/17.32	0.79	1/17.06	1.33
ST x SS	1/15.03	30.17	1/10.42	3.03

Note. N = 48. ANOVAs = analysis of variance. ST = stimulation condition. SS = set size. Smaller prior relative to the default prior = 0.15; Larger prior relative to the default prior = 1.

Supplementary Notes 7: Posterior Distribution and Sensitivity Analyses for T-Tests

Supplementary Table 8

Summary of Posterior Distribution for T-Tests

Comparison	*M*	*SD*	*LB*	*UB*
PPC & Set size 2 vs 0	0.06	0.06	0.00	0.21
PPC & Set size 4 vs 0	0.09	0.07	0.00	0.26
PPC & Set size 6 vs 0	0.07	0.06	0.00	0.22
DLPFC & Set size 2 vs 0	-0.15	0.13	-0.42	0.11
DLPFC & Set size 4 vs 0	-0.10	0.13	-0.37	0.15
DLPFC & Set size 6 vs 0	0.00	0.13	-0.26	0.27
PPC & Set size 6 vs DLPFC & Set size 6	0.07	0.06	0.00	0.23

Note. M = mean; *SD* = standard deviation; *LB* = lower bound of 95% credibility interval; *UB* = upper bound of 95% credibility interval.

Supplementary Table 9

Sensitivity of T-Tests

Stimulation	Set size	Prior					
		Small		Medium		Large	
		BF_{10}	Error (%)	BF_{10}	Error (%)	BF_{10}	Error (%)
PPC	2	1/4.12	0.00	1/9.17	0.00	1/14.40	0.00
PPC	4	1/2.48	0.00	1/5.07	0.00	1/7.81	0.02
PPC	6	1/4.46	0.00	1/10.04	0.00	1/15.79	0.00
DLPFC	2	1/1.46	0.00	1/2.46	0.02	1/3.61	0.06
DLPFC	4	1/1.86	0.00	1/3.46	0.02	1/5.21	0.07
DLPFC	6	1/2.31	0.00	1/4.66	0.02	1/7.16	0.09
PPC vs DLPFC	6	1/3.93	0.00	1/8.68	0.00	1/13.61	0.00

Note. Small prior = 0.2; Medium prior = 0.5; Large prior = 0.8.
