# Supplementary material for: TDCS over PPC or DLPFC does not improve visual working memory capacity
Source: Commun Psychol. 2024 Mar 11;2:20. doi: 10.1038/s44271-024-00067-8 (PMC11332112; doi:10.1038/s44271-024-00067-8)
Supplement: Supplementary file 3 — Reporting Summary [file 44271_2024_67_MOESM3_ESM.pdf]

nature portfolio

Corresponding author(s): Shuangke Jiang, Claudia von Bastian

Last updated by author(s): Aug 1, 2023

Reporting Summary

Nature Portfolio wishes to improve the reproducibility of the work that we publish. This form provides structure for consistency and transparency in reporting. For further information on Nature Portfolio policies, see our Editorial Policies and the Editorial Policy Checklist.

Statistics

For all statistical analyses, confirm that the following items are present in the figure legend, table legend, main text, or Methods section.

n/a	Confirmed	
☐	☒	The exact sample size (*n*) for each experimental group/condition, given as a discrete number and unit of measurement
☐	☒	A statement on whether measurements were taken from distinct samples or whether the same sample was measured repeatedly
☐	☒	The statistical test(s) used AND whether they are one- or two-sided<br>*Only common tests should be described solely by name; describe more complex techniques in the Methods section.*
☒	☐	A description of all covariates tested
☐	☒	A description of any assumptions or corrections, such as tests of normality and adjustment for multiple comparisons
☐	☒	A full description of the statistical parameters including central tendency (e.g. means) or other basic estimates (e.g. regression coefficient) AND variation (e.g. standard deviation) or associated estimates of uncertainty (e.g. confidence intervals)
☐	☒	For null hypothesis testing, the test statistic (e.g. *F*, *t*, *r*) with confidence intervals, effect sizes, degrees of freedom and *P* value noted<br>*Give P values as exact values whenever suitable.*
☐	☒	For Bayesian analysis, information on the choice of priors and Markov chain Monte Carlo settings
☒	☐	For hierarchical and complex designs, identification of the appropriate level for tests and full reporting of outcomes
☐	☒	Estimates of effect sizes (e.g. Cohen's *d*, Pearson's *r*), indicating how they were calculated

Our web collection on statistics for biologists contains articles on many of the points above.

Software and code

Policy information about availability of computer code

Data collection	Data were collected in person in our lab. The experimental task was deployed on Tatool Web (https://www.tatool-web.com) and coded using JavaScript, HTML5 and CSS.
Data analysis	All analyses were run on both R 4.1.3 and MATLAB R2021a with some analyses using the MemToolbox for MATLAB (Suchow et al. 2013). The code used to support the findings are described in mathematical and script notation in the methods section and available on OSF.

For manuscripts utilizing custom algorithms or software that are central to the research but not yet described in published literature, software must be made available to editors and reviewers. We strongly encourage code deposition in a community repository (e.g. GitHub). See the Nature Portfolio guidelines for submitting code & software for further information.

Data

Policy information about availability of data

All manuscripts must include a data availability statement. This statement should provide the following information, where applicable:

- Accession codes, unique identifiers, or web links for publicly available datasets
- A description of any restrictions on data availability
- For clinical datasets or third party data, please ensure that the statement adheres to our policy

All materials, data, experimental task code and analysis scripts are available at https://osf.io/92k4w.

Human research participants

Policy information about studies involving human research participants and Sex and Gender in Research.

Reporting on sex and gender	Participants were given a two-choice question ('Male/Female') regarding their biological gender (i.e. sex) when they filled the personal information form. Thus, sex was determined by self-report. Consent has been obtained for sharing of individual-level data. 31 females, 17 males were included in the current study. We only collected and considered sex (male vs. female) information to be consistent with the original study (Wang et al., 2019) that we replicated.
Population characteristics	A total of 48 healthy young adults were recruited (31 females, 17 males, all right-handed, 22.65 ± 4.34 years old, range 18 – 33 years). The inclusion criteria were similar to those in Wang et al. (2019): all participants had normal or corrected-to-normal vision, no metallic implant, and no history of any neurological or psychiatric illness. In addition, in the present study, only participants who were proficient in English and educated to A-level or higher were included. Furthermore, we excluded participants who self-reported that they underwent neurostimulation within the past week, were on medication with known cognitive side-effects, in particular on memory and attention, or were currently using recreational drugs (e.g., cannabis, cocaine, or methamphetamines), or were pregnant.
Recruitment	Participants were recruited through university volunteer systems, social media (e.g., Facebook), display of flyers, and word-of-mouth.
Ethics oversight	The study was approved by the University of Sheffield Research Ethics Committee (Reference number: 025631).

Note that full information on the approval of the study protocol must also be provided in the manuscript.

Field-specific reporting

Please select the one below that is the best fit for your research. If you are not sure, read the appropriate sections before making your selection.

☐ Life sciences ☒ Behavioural & social sciences ☐ Ecological, evolutionary & environmental sciences

For a reference copy of the document with all sections, see nature.com/documents/nr-reporting-summary-flat.pdf

Behavioural & social sciences study design

All studies must disclose on these points even when the disclosure is negative.

Study description	Quantitative experimental study.
Research sample	A total of 48 healthy young adults were recruited (31 females, 17 males, all right-handed, 22.65 ± 4.34 years old, range 18 – 33 years). All participants who were living in Sheffield, UK during the time of data collection. Target sample size was pre-registered on OSF (https://osf.io/n9fkp). We chose this sample size for two reasons. First, although Wang et al. (2019) reported a large effect size of Cohen's d = 1.03, yielding a (post-hoc) power of 1 - β = 0.98 for their included sample of 18 participants for analysis, simulations have shown that effect sizes are often overestimated for such small samples (Halsey et al., 2015). Therefore, we ran an a priori power analysis based on a more conservative medium effect size of Cohen's d = 0.50, a power of 1 - β = 0.90 and an α-level of 0.05, resulting in a minimum sample size of 44 (G*power 3.1; Faul et al., 2007). Second, fully counterbalancing the stimulation conditions (i.e., DLPFC, PPC and PPC/DLPFC sham) across three sessions results in 12 possible combinations; therefore, we recruited 48 healthy participants, which is a multiple of 12 and more than twice larger than the sample size in the original study.
Sampling strategy	Simple random sampling. An a priori power analysis based on a conservative medium effect size of Cohen's d = 0.50, a power of 1 - β = 0.90 and an α-level of 0.05, resulting in a minimum sample size of 44 (G*power 3.1; Faul et al., 2007)
Data collection	This lab-based study used a within-subjects, randomized and single-blinded design. All participants came to the lab for three sessions. Each session lasted about 1 hour, with an intersession-interval of at least 48 hours to allow for any possible after-effects of tDCS to return to baseline ('wash out'). Upon arrival at their first session, participants gave their written informed consent for their participation and completed a questionnaire on demographic information (age, sex, main language, handedness, and education level). At each session, participants first received the tDCS. Next, they completed short post-stimulation ratings on their current pain, attention, and fatigue levels, followed by a tDCS adverse-effects questionnaire. Next, participants completed a computerised VWM task. In addition, to measure expectation effects, at the end of the third and final session, participants were asked to guess whether they had received active or sham stimulation at each session . The computerised VWM task was deployed on Tatool Web (https://www.tatool-web.com). The questionnaires were collected using paper and pen. Only the participant and the researcher presented during each session. Participants were blind to their stimulation condition until then end of their last session.
Timing	The data collection started on 2021-07-30 and ended on 2021-11-29.
Data exclusions	No data were excluded from analyses.
Non-participation	No participants dropped out/ declined the participation.

Randomization	Within-subjects design: no group allocation is needed while participants were randomly assigned to one of the 12 possible counterbalance sequence on stimulation condition by using a generated list from Google spreadsheet.

Reporting for specific materials, systems and methods

We require information from authors about some types of materials, experimental systems and methods used in many studies. Here, indicate whether each material, system or method listed is relevant to your study. If you are not sure if a list item applies to your research, read the appropriate section before selecting a response.

Materials & experimental systems

n/a	Involved in the study
☒	☐ Antibodies
☒	☐ Eukaryotic cell lines
☒	☐ Palaeontology and archaeology
☒	☐ Animals and other organisms
☒	☐ Clinical data
☒	☐ Dual use research of concern

Methods

n/a	Involved in the study
☒	☐ ChIP-seq
☒	☐ Flow cytometry
☒	☐ MRI-based neuroimaging
